# Impact of Long-COVID on Health Care Burden: A Case Control Study

**DOI:** 10.3390/jcm12185768

**Published:** 2023-09-05

**Authors:** Bernardo Valdivieso-Martínez, Inma Sauri, Juliette Philibert, Jose Miguel Calderon, María-Eugenia Gas, Javier Diaz, Jose Luis López-Hontangas, David Navarro, Maria Jose Forner, Josep Redon

**Affiliations:** 1 The University and Polytechnic La Fe Hospital of Valencia, Avenida Fernando Abril Martorell, 106 Torre H 1st Floor, 46026 Valencia, Spain; 2 The Joint Research Unit in ICT Applied to the Reengineering of Socio-Sanitary Processes, The Medical Research Institute Hospital La Fe, Avenida Fernando Abril Martorell, 106 Torre A 7th Floor, 46026 Valencia, Spain; 3 BigData Team, INCLIVA Research Institute, University of Valencia, Menendez Pelayo, 4, 46010 Valencia, Spain; 4 Department of Microbiology, The University and Polytechnic La Fe Hospital of Valencia, 46026 Valencia, Spain; 5 Department of Microbiology, Hospital Clinico of Valencia, University of Valencia, 46010 Valencia, Spain; 6 PROVAVAC, Generalitat Valenciana, 46026 Valencia, Spain; 7 Internal Medicine, Hospital Clínico, University of Valencia, 46010 Valencia, Spain

**Keywords:** long-COVID, post-COVID, diagnostic, drug prescription, primary care, specialist, Emergency Department, case-control, propensity score

## Abstract

The objective was to identify the chronic impact of SARS-CoV-2 virus infection in new diagnostics, pharmacological prescriptions, and use of healthcare resources in patients after acute infection in a case-control study. Methods: Case-control study with observation of new diagnostics codified in the Electronic Health Recordings, with a total population of 604,000 subjects. Cases included patients diagnosed with acute infection. Matched controls in the absence of infection using a Propensity Score were also included. Observational period was 6 months. New diagnostic (CIE10), prescriptions and visits to Health Care Resources were identified. Results: 38,167 patients with a previous COVID infection and the same number of controls were analyzed. Population included < 18 years old, 7586 (mean age 10.2 years, girls 49%), and 30,581 adults (mean age 46.6 years, females 53%). In adults, 25% presented new diagnoses, while the prevalence was 16% in youth. A total of 40 new diagnostics were identified. The most frequent were diagnostics in the neuropsychiatric sphere, with older age, female, and previous admission in the Critical Care Unit being the factors related in adults, while in youth higher age was also a factor. Prescription of psychoanaleptic, psycholeptic and muscle relaxants had increased. An increment of around 20% in visits to Primary Care Physicians, Specialists and Emergency Departments was registered. Conclusion: Compared with a control group, an increment in the number of new diagnostics, new prescriptions and higher use of Health Care resources were observed. Many of the new diagnoses also occur in non-infected subjects, supporting the complex origin of so-called Long-COVID.

## 1. Introduction

The SARS-CoV-2 pandemic has severely impacted the global population, with a high morbidity and mortality rate [1]. Although attempts have been made to date to define the clinical manifestations of acute illness [2,3,4,5], beyond the first acute phase of the illness individuals with SARS-CoV-2 increase their use of healthcare resources due to persistent symptoms or new complaints [6,7,8,9,10,11,12,13,14]. Therefore, several definitions of this condition have been launched [15,16,17].

Assessment of the real impact of SARS-CoV-2 infection after the acute period is not easy due to many factors. Previous studies have analyzed possible factors related to increased healthcare demand due to persistence of previous or new ailments, which has been termed Long-COVID [9,11,13]. Even though these can be present in any infected patient, even asymptomatic, it appears that the severity of the acute infection may increase the risk [9]. Age does not appear to be a risk factor, but women were predominantly affected. The association with comorbidity, which may act as a confounding factor in the interpretation of symptoms, is unclear [18].

Analysis and estimation of the Long-COVID burden in health care have been approached from different points of view with different criteria for defining symptoms and overlapping definitions that may explain differences between the reported studies. Different time frames to define the study period and the nature of the conditions included do not contribute to clarifying the impact. In addition, multiple reports without control group or describing groups of diseases and/or symptoms with a similar clinical picture from specialized centers overestimated the impact. Few studies have analyzed the impact of a previous SARS-CoV-2 infection in the incidence of new diseases and in the Health Care burden after the acute episode during the following months [19,20]. These studies, conducted with information from Electronic Health Recordings (EHR), also used different approaches in selecting the control groups and focus on specific objectives: health care utilization [21], prevalence and incidence of symptoms after 12 weeks of acute disease and persistence of previous symptoms [22], mortality and use of health care services [23]. In addition, most studies did not include children [24,25,26].

The data obtained from EHR are capable of providing a large amount of information that can help to assess the burden of disease in terms of diagnostics, drug use and needs for Health Care resources. We used the EHR of the Valencia Community to identify the chronic impact of SARS-CoV-2 infection in our setting, reflected in the appearance of new diagnostics, new drug prescriptions, and the impact on the use of healthcare resources in patients who survived more than 30 days after the acute infection in a case-control study. Both young people and adults were included, and the control group was obtained by using Propensity Score Match (PSM).

## 2. Materials and Methods

### 2.1. Design and Participants

This was a case-control study with retrospective observation of new diagnoses coded in the EHR of two Health Care Areas of the Valencia Community, Clinic-Malvarrosa (HCA-1) and La Fe (HCA-2). The Valencia Community, ABUCASIS, is a EHR in which all subjects registered in the territory have an individual numerical code for their health status and EHR procedures associated with general medicine and public hospital areas, which guarantees the interoperability of the EHRs. Administrative data, diagnostics, all prescriptions and dispensation of subsidized treatments and hospitalization events are linked to the database that integrates all the health care interventions. The population of the Valencia Community totals more than 5 M subjects and is divided into Health Care Departments. Each has a Primary Care Area and a Large Hospital of reference. In the present study, data included two different Departments, which cover a total of 604,000 subjects.

### 2.2. Legal and Ethical Procedures

Data collection and analysis has been carried out taking into account all the necessary elements to safeguard patient privacy by means of a double-layer methodology of pseudo-anonymization by the health authorities and subsequent anonymization prior to analysis. The derived information is managed as aggregated data. The databases have been stored in the facilities of the INCLIVA Research Institute facilities and in La Fe Research Institute, in servers with the usual protection standards implemented to prevent access by third parties. The research is carried out in full compliance with the provisions of Regulation (EU) 2016/679 of the European Parliament and of the Council of 27 April 2016 on the protection of individuals with regard to the processing of personal data. Likewise, compliance with the 17th additional provision on the processing of health data of Organic Law 3/2018 of 5 December on the Protection of Personal Data and the guarantee of digital rights and the applicable sectoral legislation is guaranteed. The informed consent exemption has been requested and obtained. The information was available for research, anonymized in accordance with the Spanish Law on Data Protection, and with the approval for its study by the Committee for Ethics and Clinical Trials of the Hospitals Clinico of Valencia and La Fe. Spanish Law 3/2018 on Data Protection and the Guarantee of Digital Rights and corresponding European norms (GDPR) [27] were followed.

### 2.3. Subjects and Procedures

Cases included patients of both sexes, diagnosed with acute SARS-CoV-2 infection by PCR or antigen in the Microbiology Lab of the hospitals, regardless of whether the patients were hospitalized (admitted) or not. The same number of matched controls in absence of SARS-CoV-2 infection were also included. Population has been divided into youth, less than 18-years-old, and adults.

Case-control data were obtained from three periods:

*Period A*, case selection time of patients with SARS-CoV-2 infection from March 2020 until 31 July 2021.

*Period B*, from 1 July to 31 December 2019, in which diseases and medications prescribed to cases with SARS-CoV-2 infection and controls prior to infection were recorded.

**Figure 1:**
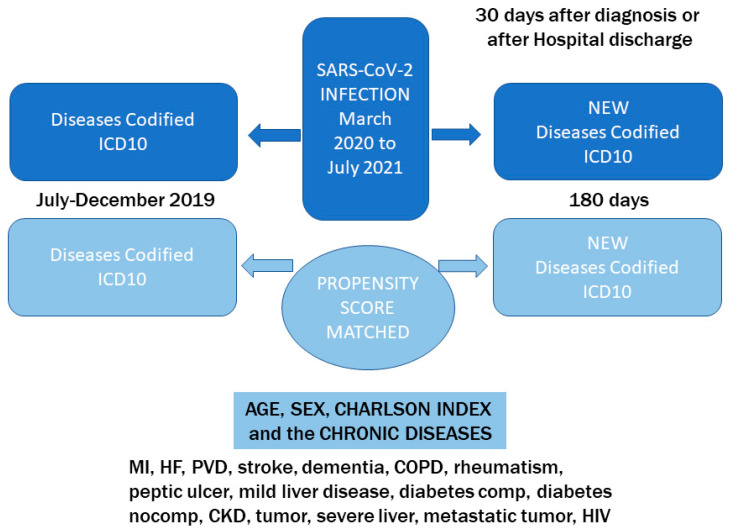
Study design.

*Period C*, 30 days from the date of diagnosis, in those seen in primary care or after hospital discharge up to 6 months later, for a total of 180 days, in which diseases and medications registered in the EHR system, not present before the infection period (period B), were censored (Figure 1).

For each subject, patients or control, the following data were obtained: age, sex, hospital admission due to COVID. New diagnoses and prescriptions were considered when they had not been previously recorded in period B. Information on previous diagnosis, period B, and new diagnosis, Period C, were obtained from CIE-10 and medication from the ATC code recorded in the EHR. CIE-10 codes obtained during period B in subjects that had SARS-CoV-infection were used for PSM selection of the control group.

During period C, the Health Resources used (number of patients and visits to Primary Care Physicians, Specialists and Emergency room) were obtained from administrative data from Primary Care Health Care Centers, Hospital Outpatient Clinics and Emergency Departments, where all visits to the Primary Physicians (Health Care Centers), Specialists (Hospital Outpatients Clinics) and the Emergency room are automatically registered in the digital system. Hospitalizations and mortality during period C were also identified in the Medical Records of hospitalization.

### 2.4. Statistical Analysis

Data are provided as absolute numbers with concomitant percentages. Continuous measurements were provided as means and standard deviation. Incidence per 100 patients/6 months was calculated. Difference between the number and incidence of new diagnoses were calculated by using unpaired student t and Chi-squared. Factors in the incidence of new diseases in the different systems are assessed by using logistic regression.

To select the control group, a PSM process included: age, sex, Charlson index and all the chronic diseases of the index (chronic diseases present before the period of pandemic, which include myocardial infarction, heart failure, peripheral artery disease, stroke, dementia, chronic obstructive pulmonary disease, rheumatism, peptic ulcer, liver disease, diabetes, chronic kidney disease, tumor, metastatic tumor, HIV infection) and the time period of the corresponding case.

Furthermore, sensitivity analysis was performed given the separate information from the two HCAs and compared between them. Statistical analysis was performed by using R 6.3.1.

The funders of the study had no role in study design, data collection, data analysis, data interpretation or at the time to writing of the reports.

## 3. Results

### 3.1. General Characteristics

A total of 38,167 patients (mean age 40.3 yr [21.9], 53% female) with previous COVID diagnosed by positive SARS-CoV-2 RT-PCR or antigen test, and the same number of controls were analyzed. Population included youth < 18 years old, 7586 (mean age 10.2 yr, girls 49%), and 30,581 adults (mean age 46.6 yr, females 53%). The general characteristics of the study population, patients and their respective controls are shown in Table 1a,b for adults and youth, respectively. The maximum follow-up time was 176 days after 30 days of confirming infection. The total number of patients hospitalized due to acute SARS-CoV-2 infection was 3133 (8%) adults and 50 (0.06%) youths. Men were more frequently hospitalized due to SARS-CoV-2 infection than females, 5.0% and 2.5%, respectively (*p* < 0.001). The most prevalent chronic diseases were COPD, diabetes and neoplasm in adults and asthma in youth. The averages for Charlson index were 0.56 ± 1.07 and 0.45 ± 0.89, respectively, in each of the two HCAs.

### 3.2. New Diagnostics

The new diagnostics recorded in the EHR in SARS-CoV-2 patients and controls with the corresponding CIE-10 code are shown in Table 2a,b. Among the total cases in adults, 30,581, 25% presented new diagnoses, while the prevalence was 16% in youths. Number of subjects, percentage of the differences between patients and controls, incidence of the disease per 100 patients for the six months in the two HCAs, are shown in Table 2a,b, for adult and youth, respectively. In patients with previous SARS-CoV-2 infection, a total of 40 presenting new diagnostics, in more than 20 subjects each, were identified. The new diagnostics in adults were Neuropsychiatric 10, Respiratory, Musculoskeletal and Dermatology 5, Digestive tract, Gynecology and Infectious disease 4, Cardiovascular 2 and Hematology, Ophthalmology and Oral cavity, 1 each. The number of diagnostics and the number of patients for each system in adults and youth, in the two HCAs, are shown in Supplementary Table S1a,b.

**Figure 2:**
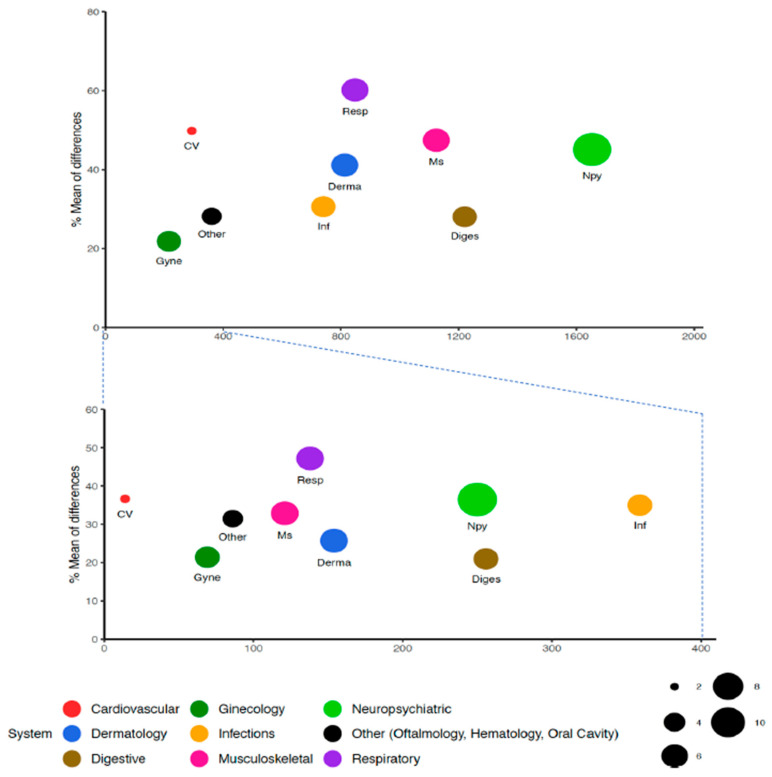
Average of differences in new diagnostics between previous SARS-CoV-2 infection and controls for the different systems affected and the number of subjects in each in both youth (**lower panel**) and adult (**upper panel**). The colors of the bullets represent the different systems affected. The size of bullets represent the number of different symptoms identified in each system. X axis shows the number of subjects affected.

Overall, the most common diseases identified in adults were functional dyspepsia, dizziness and giddiness, unspecified abdominal pain, weakness, headache, anxiety and low back pain. and the most prevalent in patients over controls were anosmia, depressed mood, weakness, fatigue, hair loss, unspecified dyspnea and myalgia. In youths, pharyngitis, tonsillitis, unspecified fever, abdominal pain and cough were most prevalent, but in more than 50%, as compared to control, were weakness and conditions with a very low incidence. Figure 2 represent the average of differences between COVID patients and controls for the different systems and the number of subjects in each for both youth and adult. Some patients presented symptoms pertaining not only to one system. Diagnostics in more than three systems in the same patient were observed in 1%, affecting three systems 3.4%, and two in 17%. A total of 638 (1.1%) deaths were recorded, 148 among those with infection and 290 in controls.

As a sensitivity test, differences between the two HCAs, corresponding to the percentage of diseases higher in the COVID group as compared to controls, ranged from 14 diagnostics with less than 5% differences to 9 diagnostics with more than 20% differences.

### 3.3. Factors Related to the Most Common Affected Systems

The factors related to the systems affected, Neuropsychiatric, Respiratory, Muscle skeletal and Infectious diseases were calculated by using logistic regression, including age, sex, Charlson index, hospitalization in the acute episode and CCU admission during the SARS-CoV-2 infection. In adults: for Neuropsychiatric diagnoses, age (OR 1.01, 95 CI 1.00–1.02; *p* = 0.007), females (OR 1.87, 95 CI 1.61–2.16; *p* < 0.001) and UCI admission (OR 2.16, 95 CI 1.06–4.43; *p* = 0.04) were the most determinant; for Respiratory, age (OR 1.01, 95 CI 1.00–1.02; *p* = 0.002), females (OR 1.50, 95 CI 1.22–1.85; *p* < 0.001) and Charlson index (OR 1.14, 95 CI 1.06–1.23; *p* = 0.001); for Muscle skeletal, females (OR 1.89, 95 CI 1.57–2.25; *p* < 0.001) and Charlson index (OR 1.09, 95 CI 1.01–1.17; *p* = 0.03); for Infections, age (OR 1.89, 95 CI 1.57–2.25; *p* = 0.004); females (OR 1.57, 95 CI 1.28–1.92; *p* < 0.001), Charlson index (OR 1.13, 95 CI 1.04–1.23; *p* = 0.003) and previous hospitalization (OR 1.47, 95 CI 1.06–2.04; *p* = 0.02). In youth only age was significant; in the oldest, Neuropsychiatric (OR 1.06, 95 CI 1.02–1.10; *p* = 0.003) and Musculoskeletal (OR 1.14, 95 CI 1.08–1.21; *p* < 0.001); and in the younger patients, Respiratory (OR 0.88, 95 CI 0.84–0.93; *p* < 0.001) and Infectious disease (OR 0.94, 95 CI 0.92–0.97; *p* < 0.001), mainly of the respiratory tract.

### 3.4. New Drug Prescription

The number of new drugs (AT2) prescribed to COVID patients and controls during the observational period are shown in Table 3a,b. The percentage of increment between cases and controls in adults was superior to 14% in all of them, except for analgesics, more frequent in controls. In youth, the number of new treatments was small, although pre-scription of psychoanaleptics was more frequent, as well as cough and cold preparation. The factors related to the increment of prescriptions of psychoanaleptics were age (OR 1.009, 95 CI 1.002–1.016; *p* = 0.02), females (OR 1.74, 95 CI 1.31–2.31; *p* < 0.001) and previous hospitalization (OR 2.70, 95 CI 1.84–3.97; *p* < 0.001) and for psycholeptics only previous hospitalization (OR 1.58, 95 CI 1.03–2.42; *p* = 0.02), and the same for muscle relaxants (OR 1.92, 95 CI 1.17–3.13; *p* = 0.01).

### 3.5. Utilization of Health Care Resources

The Health Care resources used, number of patients and visits to Primary Care, Specialist clinics, Emergency room and Hospital admissions for the cases and controls are shown in Table 4a,b. Although there are differences between the two HCAs, overall, the use of health care services was more frequent among the COVID patients as compared to the controls in both HCAs and in the adult and youth groups. The increment of the services requested were both for visits within the Primary Care Physicians and Specialists, as well as attending Emergency Departments of the hospitals. Concerning the necessity for hospital admission, the increment in the COVID patients as compared to controls was small and other factors such as prescheduled admission for surgery impact the numbers. The factors related to the increment of use of health resources were for Primary Care: age (OR 1.03, 95 CI 1.02–1.04; *p* < 0.001), females (OR 1.57, 95 CI 1.46–1.70; *p* < 0.001), Charlson index (OR 1.46, 95 CI 1.37–1.56; *p* < 0.001) and previous hospitalizations: (OR 1.82, 95 CI 1.42–2.33; *p* < 0.001). For demand in Emergency room: females (OR 1.21, 95 CI 1.11–1.31; *p* < 0.001), Charlson index (OR 1.27, 95 CI 1.22–1.31; *p* < 0.001) and previous hospitalizations: (OR 1.81, 95 CI 1.57–2.08; *p* < 0.001).

## 4. Discussion

The Long-COVID condition, the persistence of symptoms presented during the acute phase and new ones developed afterwards, have received increasing attention due to the lack of knowledge of the causal mechanisms, the impact on quality of life, the uncertainties in its prognosis and the overload on health systems. In the present study, following the CDC definition [15], we evaluated the increase in diagnoses and the use of health care resources in the post-COVID in patients from two HCAs who not only have different physicians in primary care but also in different referral hospitals. Compared to a control group, carefully selected by PSM, an increase in the number of new diagnoses, new prescriptions and increased use of Health Care resources was observed. Although the increment was also observed in youth, their proportion was lower as compared to adults.

The study design attempted to avoid a potential bias. All patients included were diagnosed based on reverse-transcription-polymerase chain reaction or antigen test in the Hospital Microbiology Departments. The observational period started 30 days after diagnosis of the acute infection or 30 days after hospital discharge, in order to minimize the persistence of mild symptoms. In both groups of patients, infected and controls, the existence of previous diagnostics and pharmacological treatments in the six months before the infection were identified. Likewise, no vaccination was recorded in the patients or controls during the study period. The selection of a control group is mandatory to evaluate the impact of viral infection per se in the use of Health Care resources, since many of the new diagnostics or symptoms are not virus-induced and could be a consequence of stress, fear, and/or lockdown conditions, which have impacted infected and non-infected patients to a greater or lesser extent. 

The present study was performed in patients infected with the SARS-CoV-2 residing in Health Care areas with similar organizations in both Primary Care and Hospital resources. The inclusion of the two HCAs not only allows study of a large number of patients but also observation of potential differences in new diagnostics and prescriptions derived from clinical routine to avoid possible biases. Forty new diagnoses, absent before the infection period, were the most frequent of those identified in the infected patients. The most frequent in adults were those belonging to the Neuropsychiatric sphere, although Musculoskeletal, Respiratory, Digestive and Dermatologic diagnoses were also present, the more frequent diagnostics being: functional dyspepsia, dizziness and giddiness, unspecified abdominal pain, weakness, headache, anxiety, and low back pain. In general, the most frequent diagnostics coincided with those described in the literature [28,29,30,31,32,33,34,35,36,37] although in the controls the prevalence was also high in the present study. In fact, upper and lower Digestive tract symptoms have similarities with postinfectious functional dyspepsia and irritable bowel syndrome [28], as well as with mental health symptoms [31,35,36]. In contrast, the most prevalent by far in patients relative to controls were anosmia, depressed mood, weakness, fatigue, hair loss, unspecified dyspnea and myalgia [29,32,37]. These more specific symptoms have a relatively low frequency with the exception of weakness [35] and unspecified dyspnea [37]. The factors most frequent related to the development of new diagnoses were older age, females, previous hospitalization and Charlson index, mimicking that reported in previous publications [9,10,11,12,13].

Long-COVID in children and adolescents has been reported in a large number of studies. However, metanalysis [24,25] of studies reporting long COVID in this age group concluded that the studies published are heterogeneous in design, in the way of collecting information and even in the diagnostic method used to define acute infection, taking into account serologic data [25]. An attempt to define criteria to diagnose Long COVID in children derived from a Delphi process does not contribute to more precision [38]. A review published in November 2021 identified 214 articles reporting a large variability in prevalence, ranging from 1.6% to 70%. In the present study, one of the largest published, prevalence was 16%. This age group appears to be less susceptible than adults, but the reasons for this have not yet been fully understood, although they could be explained by fewer comorbidities and milder symptoms that do not require hospitalization. Infectious disease, mainly in the pharynx and upper respiratory tract, headache, unspecified skin lesions, abdominal pain and diarrhea were the most common, but headache and anxiety were also present. Even though the percentage of diagnosis in COVID infection was greater as compared to control, overall the numbers are small, and some reports did not find differences between infected and controls [39]. The reason that, for many of the new symptoms, there are hardly any differences between cases and controls may be due to the impact of lockdown and/or fear of infection in the population. Regarding age groups, some studies reported that Long-COVID was more frequent in those 6 to 11 years old [39]. In our large youth group, prevalence is 16% less frequent than in adults, and the higher the age, the higher the risk for neuropsychiatric and musculoskeletal issues, while respiratory and infections predominate in the youngest. 

It is worth commenting that, despite new diagnoses, including also those from specialists and/or hospital admission, myocardial infarction, peripheral venous thrombosis, and pulmonary embolism were low, with less than 20 cases each in COVID patients, and no systemic inflammatory syndrome was registered.

No new prescriptions were reported in the majority of publications [9]. As for the new pharmacological treatments, the most commonly used were psycholeptics, anxiolytics, and psychoanaleptics, antidepressants, followed by anti-inflammatory, antithrombotic, pain medication and muscle relaxants. However, many of these classes, anxiolytics, anti-inflammatories and antithrombotics were also widely prescribed in controls, but the big differences were in psychoanaleptics, although psycholeptics and anti-inflammatory were also prescribed. 

Finally, we analyzed the impact on the overall burden of care compared to the needs of controls and documented an excess health care burden with patients in the 6 months after the acute stage of infection. A higher number of infected patients not only demanded more frequent care by primary and specialty care physicians, but also a higher number of consultations. Likewise, the number of Emergency Department visits and hospitalizations increased significantly. Similar data was observed in a large population study in Canada [40]. The observed increment in burden of care between cases and controls occurred in both adults and youth, even though the figures in children were lower.

Several models of pathogenesis have been proposed to explain the persistence of symptoms or new diagnoses. Persistence of the virus or a virus component [41] which exacerbated immune response, resulting in increased levels of pro-inflammatory cytokines, could explain organ damage and prolonged symptoms, such as fatigue, headache, and smell impairment [41,42]. In addition, a mechanism of molecular mimicry between autoantigens and spike epitopes has been proposed [43]. However, many of the sequelae in Long COVID are difficult to distinguish between functional complaints which are viral-driven and social restriction effects. Finally, the potential impact of vaccination has not been contemplated. The reason is that all infections occur before January 2021, when vaccination begins in Spain. Regarding vaccination during the six months of follow-up in controls, a small percentage may have received the vaccine after January 2021. Furthermore, the vaccines administered came from different kind of vaccines, making it not possible to estimate the real impact in new diagnostics.

The strengths and limitations of the study should be contemplated. Assessment of new diagnostics in youths and adult cases collected from the general population and propensity score matched controls allowed gauging the impact of the COVID-19 infection. Not only the selection of cases and controls but also the identification of previous diagnosis and medications offered robust data to compare for assessing new diagnostics and treatments. The study does not contemplate a clinical evaluation of the new diagnostic, but the objective of the study was not to characterize the new diagnostics. Finally, limitations also include all those inherent to the EHR, although we tried to minimize these and only included patients with the necessary records in the analysis.

In conclusion, from a routine care case-control study conducted with EHR information, a large increase in the frequency of new diagnoses was observed, together with an increase in the use of medication and Health Care services. Many of the new diagnoses also occur in non-infected subjects, supporting the complex origin of so-called Long-COVID, in which the virus itself is not the origin of many of the reported symptoms and conditions. Scalable and integrative healthcare models comparing physical and mental health of long-term survivors of COVID-19 should be developed.

## Figures and Tables

**Table 1:** (**a**). General characteristics of study population cases and controls, >17-year-old, in the two Centers. (**b**). General characteristics of study population cases and controls, <18-year-old, in the two Centers.

(a)				
	CENTER A		CENTER B	
	Number COVID	Number Controls	Number COVID	Number Controls
Number	16,382	16,389	14,199	14,214
Age (yr)	47.63 ± 17.73	47.65 ± 17.73	45.93 ± 17.10	45.90 ± 17.12
Female (%)	8861 (54.1%)	8860 (54.1%)	7426 (52.3%)	7433 (52.3%)
Time of observation (days)	176.06 ± 20.53	176.05 ± 20.53	179.00 ± 7.68	179.03 ± 7.35
**Care for COVID infection**				
Hospital (ns)	1726	116	1357	53
Charlson_index	0.63 ± 1.16	0.63 ± 1.16	0.51 ± 0.96	0.51 ± 0.96
**Chronic disease: (ns)**				
Acute myocardial infarction	191 (1.2%)	192 (1.2%)	78 (0.5%)	78 (0.5%)
Congestive Heart Failure	255 (1.6%)	255 (1.6%)	121 (0.9%)	121 (0.9%)
Peripheral Vascular Disease	292 (1.8%)	292 (1.8%)	140 (1.0%)	141 (1.0%)
Stroke	309 (1.9%)	308 (1.9%)	169 (1.2%)	169 (1.2%)
Dementia	130 (0.8%)	131 (0.8%)	144 (1.0%)	144 (1.0%)
Chronic Pulmonary Disease	2801 (17.1%)	2801 (17.1%)	2004 (14.1%)	2003 (14.1%)
Rheumatic disease	200 (1.2%)	200 (1.2%)	138 (1.0%)	138 (1.0%)
Peptic ulcer	379 (2.3%)	380 (2.3%)	292 (2.1%)	292 (2.1%)
Mild liver disease	684 (4.2%)	685 (4.2%)	587 (4.1%)	587 (4.1%)
Diabetes without complications	1542 (9.4%)	1542 (9.4%)	1101 (7.7%)	1101 (7.7%)
Diabetes with complications	272 (1.7%)	272 (1.7%)	182 (1.3%)	182 (1.3%)
Hemiplegia	62 (0.4%)	62 (0.4%)	50 (0.4%)	53 (0.4%)
Chronic kidney disease	362 (2.2%)	362 (2.2%)	277 (1.9%)	277 (1.9%)
Neoplasm	953 (5.8%)	953 (5.8%)	627 (4.4%)	626 (4.4%)
Moderate or severe liver disease	22 (0.1%)	21 (0.1%)	3 (0.0%)	3 (0.0%)
Metastatic tumor	50 (0.3%)	50 (0.3%)	33 (0.2%)	33 (0.2%)
HIV	40 (0.2%)	40 (0.2%)	45 (0.3%)	45 (0.3%)
(**b**)				
	**CENTER A**		**CENTER B**	
	**Number COVID**	**Number Controls**	**Number COVID**	**Number Controls**
Number	3981	3974	3605	3590
Age (yr)	10.23 ± 4.99	10.21 ± 4.99	9.42 ± 5.19	9.39 ± 5.18
Female (%)	1956 (49.1%)	1957 (49.2%)	1783	1778
Time of observation (days)	179.11 ± 4.21	179.12 ± 4.18	179.26 ± 3.89	179.23 ± 4.69
**Care for COVID infection**				
Hospital (ns)	23	4	27	4
Average Charlson_index	0.29 ± 0.52	0.29 ± 0.52	0.22 ± 0.45	0.22 ±0.45
**Chronic disease: (ns)**				
Acute myocardial infarction	1 (0%)	0 (0%)	0 (0%)	0 (0%)
Congestive Heart Failure	2 (0.1%)	2 (0.1%)	1 (0%)	1 (0%)
Peripheral Vascular Disease	0 (0%)	0 (0%)	3 (0.1%)	2 (0.1%)
Stroke	36 (0.9%)	37 (0.9%)	20 (0.6%)	20 (0.6%)
Dementia	1 (0%)	0 (0%)	0 (0%)	0 (0%)
Chronic Pulmonary Disease	947 (23.8%)	947 (23.8%)	681 (18.9%)	682 (19.0%)
Rheumatic disease	1 (0%)	1 (0%)	1 (0%)	1 (0%)
Peptic ulcer	15 (0.4%)	14 (0.4%)	6 (0.2%)	6 (0.2%)
Mild liver disease	15 (0.4%)	14 (0.4%)	4 (0.1%)	4 (0.1%)
Diabetes without complications	6 (0.2%)	6 (0.2%)	10 (0.3%)	10 (0.3%)
Diabetes with complications	2 (0.1%)	2 (0.1%)	2 (0.1%)	2 (0.1%)
Hemiplegia	5 (0.1%)	5 (0.1%)	9 (0.2%)	6 (0.2%)
Chronic kidney disease	2 (0.1%)	2 (0.1%)	2 (0.1%)	2 (0.1%)
Neoplasm	53 (1.3%)	53 (1.3%)	18 (0.5%)	19 (0.5%)
Moderate or severe liver disease	0 (0%)	1 (0%)	0 (0%)	0 (0%)
Metastatic tumor	1 (0%)	1 (0%)	0 (0%)	0 (0%)
HIV	0 (0%)	0 (0%)	0 (0%)	0 (0%)

**Table 2:** (**a**). New diagnostics recorded in the study population >17-year-old in the two Centers. (**b**). New diagnostics recorded in the study population <18-year-old in the two Centers.

(a)								
	CENTER A				CENTER B			
CIE 10. Code and Description	Number COVID	Number Control	%	Incidence 100 Pat/6 Months	Number COVID	Number Control	%	Incidence 100 Pat/6 Months
**Total number**	**3843**	**2540**	**0.34**		**2883**	**1715**	**40.51**	
**NEUROPSYCHIATRIC**								
F32.9-Major depressive disorder. single episode. unspecified	72	52	27.78	0.48	67	24	64.18	0.48
F34.1-Dysthymic disorder	45	34	24.44	0.30	47	27	42.55	0.33
F41.9-Anxiety disorder. unspecified	169	127	24.85	1.53	151	105	30.46	1.08
F43.20-Adjustment disorder. unspecified	24	15	37.50	0.15	31	10	67.74	0.22
F43.21-Adjustment disorder with depressed mood	20	8	60.00	0.13	18	6	66.67	0.13
G43.909-Migraine. unspecified. not intractable. without status migraine.	55	29	47.27	0.37	49	32	34.69	0.35
G47.00-Insomnia. unspecified	144	112	22.22	1.06	132	80	39.39	0.94
R42-Dizziness and giddiness	205	133	35.12	1.56	152	91	40.13	1.08
R43.0-Anosmia	36	5	86.11	0.23	46	4	91.30	0.33
R51-Headache	158	117	25.95	1.23	166	86	48.19	1.18
**INFECTIOUS**								
B02.9-Zoster without complications	62	37	40.32	0.41	34	26	23.53	0.24
J02.9-Acute pharyngitis. unspecified	190	138	27.37	1.31	116	63	45.69	0.82
J03.90-Acute tonsillitis. unspecified	99	67	32.32	0.65	58	39	32.76	0.41
R50.9-Fever. unspecified	86	83	3.49	0.58	115	64	44.35	0.82
**DERMATOLOGIC**								
L29.9-Pruritus. unspecified	44	40	9.09	0.28	57	34	40.35	0.40
L30.9-Dermatitis. unspecified	113	62	45.13	0.72	84	57	32.14	0.60
L50.9-Urticaria. unspecified	60	34	43.33	0.41	51	43	15.69	0.36
L65.9-Nonscarring hair loss. unspecified	104	26	75.00	0.69	110	8	92.73	0.78
L98.9-Disorder of the skin and subcutaneous tissue. unspecified	128	79	38.28	0.83	100	65	35.00	0.71
**RESPIRATORY**								
J12.89-Other viral pneumonia	16	1	93.75	0.11	14	2	85.71	0.10
R05-Cough	120	63	47.50	0.87	117	53	54.70	0.83
R06.00-Dyspnea. unspecified	118	49	58.47	0.78	157	36	77.07	1.12
R07.89-Other chest pain	92	65	29.35	0.60	111	43	61.26	0.79
R07.9-Chest pain. unspecified	64	40	37.50	0.44	92	42	54.35	0.65
**DIGESTIVE**								
K21.9-Gastro-esophageal reflux disease without esophagitis	79	57	27.85	0.55	50	29	42.00	0.35
K30-Functional dyspepsia	270	208	22.96	2.72	248	130	47.58	1.77
R10.9-Unspecified abdominal pain	170	130	23.53	1.22	174	146	16.09	1.24
R19.7-Diarrhea. unspecified	148	129	12.84	1.05	145	85	41.38	1.03
**GYNECOLOGY**								
N91.2-Amenorrhea. unspecified	28	18	35.71	0.18	20	14	30.00	0.14
N92.1-Excessive and frequent menstruation with irregular cycle	25	19	24.00	0.16	38	28	26.32	0.27
N92.6-Irregular menstruation. unspecified	17	19	−11.76	0.11	34	13	61.76	0.24
N94.6-Dysmenorrhea. unspecified	31	28	9.68	0.21	30	28	6.67	0.21
**MUSCULOSKELETAL**								
M54.5-Low back pain	184	159	13.59	1.73	172	145	15.70	1.23
M54.9-Dorsalgia. unspecified	92	60	34.78	0.65	91	55	39.56	0.65
M79.1-Myalgia	80	39	51.25	0.52	91	41	54.95	0.65
R53.1-Weakness	191	62	67.54	1.30	174	63	63.79	1.24
R53.83-Other fatigue	46	16	65.22	0.30	42	11	73.81	0.30
**OPHTHALMOLOGY**								
H10.9-Unspecified conjunctivitis	69	52	24.64	0.44	75	56	25.33	0.53
**HEMATOLOGIC**								
D64.9-Anemia. unspecified	69	51	26.09	0.48	90	50	44.44	0.64
**CARDIOVASCULAR**								
R00.0-Tachycardia. unspecified	51	34	33.33	0.33	48	16	66.67	0.34
R00.2-Palpitations	42	24	42.86	0.27	52	24	53.85	0.37
**ORAL CAVITY**								
K12.0-Recurrent oral aphthae	27	19	29.63	0.18	32	25	21.88	0.23
(**b**)								
	**CENTER A**				**CENTER B**			
**CIE 10. Code and Description**	**Number COVID**	**Number Control**	**%**	**Incidence 100 Pat/6 Months**	**Number COVID**	**Number Control**	**%**	**Incidence 100 Pat/6 Months**
**Total number**	**801**	**644**	**0.20**		**584**	**387**	**33.73**	
**NEUROPSYCHIATRIC**								
F32.9-Major depressive disorder. single episode. unspecified	6	4	33.33	0.15	5	2	60.00	0.14
F34.1-Dysthymic disorder	4	2	50.00	0.10	1	2	−100.0	0.03
F41.9-Anxiety disorder. unspecified	34	28	17.65	0.91	28	17	39.29	0.78
F43.20-Adjustment disorder. unspecified	2	1	50.00	0.05	6	1	83.33	0.17
F43.21-Adjustment disorder with depressed mood	2	3	−50.00	0.05	3	1	66.67	0.08
G43.909-Migraine. unspecified. not intractable. without status migraine.	7	8	−14.29	0.18	11	3	72.73	0.31
G47.00-Insomnia. unspecified	2	2	0.00	0.05	7	3	57.14	0.20
R42-Dizziness and giddiness	22	13	40.91	0.59	30	15	50.00	0.84
R43.0-Anosmia	3	2	33%	0.08	5	1	80.00	0.14
R51-Headache	47	45	4.26	1.41	47	29	38.30	1.32
**INFECTIOUS**								
B02.9-Zoster without complications	3	1	66.67	0.08	2	0	100.0	0.06
J02.9-Acute pharyngitis. unspecified	83	69	16.87	2.42	30	29	3.33	0.84
J03.90-Acute tonsillitis. unspecified	76	66	13.16	2.14	44	23	47.73	1.23
R50.9-Fever. unspecified	79	67	15.19	2.71	75	62	17.33	2.11
**DERMATOLOGIC**								
L29.9-Pruritus. unspecified	5	5	0.00	0.13	6	8	−33.33	0.17
L30.9-Dermatitis. unspecified	31	29	6.45	0.81	22	13	40.91	0.61
L50.9-Urticaria. unspecified	24	15	37.50	0.68	16	12	25.00	0.45
L65.9-Nonscarring hair loss. unspecified	5	2	60.00	0.13	7	2	71.43	0.20
L98.9-Disorder of the skin and subcutaneous tissue. unspecified	25	21	16.00	0.66	21	14	33.33	0.59
**RESPIRATORY**								
J12.89-Other viral pneumonia	12	11	10.00	0.21	10	10	0.00	0.21
R05-Cough	49	33	32.65	1.64	47	26	44.68	1.32
R06.00-Dyspnea. unspecified	3	1	66.67	0.08	9	0	100.00	0.25
R07.89-Other chest pain	6	1	83.33	0.15	11	5	54.55	0.31
R07.9-Chest pain. unspecified	10	4	60.00	0.26	10	7	30.00	0.28
**DIGESTIVE**								
K21.9-Gastro-esophageal reflux disease without esophagitis	4	2	50.00	0.10	6	3	50.00	0.17
K30-Functional dyspepsia	17	21	−23.53	0.43	25	15	40.00	0.70
R10.9-Unspecified abdominal pain	65	54	16.92	2.09	72	54	25.00	2.03
R19.7-Diarrhea. unspecified	41	46	−12.20	1.32	41	32	21.95	1.15
**GYNECOLOGY**								
N91.2-Amenorrhea. unspecified	5	5	0.00	0.13	4	2	50.00	0.11
N92.1-Excessive and frequent menstruation with irregular cycle	2	2	0.00	0.05	3	2	33.33	0.08
N92.6-Irregular menstruation. unspecified	8	10	−25.00	0.21	9	5	44.44	0.25
N94.6-Dysmenorrhea. unspecified	19	15	21.05	0.50	21	11	47.62	0.59
**MUSCULOSKELETAL**								
M54.5-Low back pain	19	10	47.37	0.50	15	13	13,33	0,42
M54.9-Dorsalgia. unspecified	11	12	−9.09	0.29	8	7	12.50	0.22
M79.1-Myalgia	9	6	33.33	0.24	10	8	20.00	0.28
R53.1-Weakness	22	8	63.64	0.58	17	8	52.94	0.47
R53.83-Other fatigue	11	5	54.55	0.28	5	3	40.00	0.14
**OPHTHALMOLOGY**								
H10.9-Unspecified conjunctivitis	19	16	15.79	0.50	24	18	25.00	0.67
**HEMATOLOGIC**								
D64.9-Anemia. unspecified	5	3	40.00	0.13	11	6	45.45	0.31
**CARDIOVASCULAR**								
R00.0-Tachycardia. unspecified	2	2	0.00	0.05	3	2	33.33	0.06
R00.2-Palpitations	5	1	80.00	0.13	3	2	33.33	0.08
**ORAL CAVITY**								
K12.0-Recurrent oral aphthae	9	7	22.22	0.25	20	12	40.00	0.56

**Table 3:** (**a**). New prescriptions (Anatomical Therapeutic Chemical code) recorded in the study population >17-year-old in the two Centers. (**b**). New prescriptions (Anatomical Therapeutic Chemical code) recorded in the study population <18 year-old in the two Centres.

(a)						
	CENTER A			CENTER B		
	Number COVID	Number Control	%	Number COVID	Number Control	%
N05-PSYCHOLEPTICS	225	184	18%	285	205	28%
N06-PSYCHOANALEPTICS	199	126	37%	207	123	41%
M01-ANTI-INFLAMMATORY	139	119	14%	237	230	3%
B01-ANTITHROMBOTIC	180	138	23%	136	106	22%
R03-ASTHMA TREATMENT	152	64	58%	121	62	49%
B03-ANTIANEMIC	155	96	38%	226	140	38%
M03-MUSCLE RELAXANTS	149	108	28%	197	127	36%
N02-ANALGESICS	97	124	−28%	214	201	6%
C09-ARBs.	112	71	37%	128	92	28%
G03-SEX HORMONES	78	45	42%	75	43	43%
A04-ANTIEMETICS AND ANTINAUSEANTS	87	54	38%	50	43	14%
R05-COUGH AND COLD PREPARATIONS	61	32	48%	49	27	45%
(**b**)						
	**CENTER A**			**CENTER B**		
	**Number COVID**	**Number Control**	**%**	**Number COVID**	**Number Control**	**%**
N05-PSYCHOLEPTICS	31	27	13%	42	34	19%
N06-PSYCHOANALEPTICS	20	7	65%	15	11	27%
M01-ANTI-INFLAMMATORY	59	62	−5%	90	70	22%
B01-ANTITHROMBOTIC	8	8	0%	8	3	63%
R03-ASTHMA TREATMENT	32	26	19%	46	31	33%
B03-ANTIANEMIC	14	12	14%	36	21	42%
M03-MUSCLE RELAXANTS	4	1	75%	5	7	−40%
N02-ANALGESICS	29	24	17%	50	58	−16%
C09-ARBs.	0	1		0	0	
G03-SEX HORMONES	22	9	59%	15	11	27%
A04-ANTIEMETICS AND ANTINAUSEANTS	3	2	33%	2	0	
R05-COUGH AND COLD PREPARATIONS	27	16	41%	21	10	52%

**Table 4:** (**a**). Burden of Health Care resources in the study population >17-year-old in the two Centers. (**b**) Burden of Health Care resources in the study population <18-year-old in the two Centers.

(a)						
	CENTER A			CENTER B		
	Number COVID	Number Control	%	Number COVID	Number Control	%
**Number**	16,382	16,389		14,199	14,214	
**Primary Care Visits**						
Number of visits	94,131	76,702	19%	76,902	56,563	26%
Number of patients	14,074	12,967	8%	11,896	9905	17%
**Specialist Visits**						
Number of visits	21,439	16,255	24%	19,892	13,540	32%
Number of patients	6389	5106	20%	5331	3720	30%
**Emergency Room**						
Number of visits	3503	2746	22%	2727	1706	37%
Number of patients	2270	1838	19%	1885	1227	35%
**Hospital Admissions**						
Number of admissions	838	792	5%	603	438	27%
Number of patients	672	641	5%	518	366	29%
Admision from Emergency	417	476	−14%	318	208	35%
Admision scheduled	196	160	18%	110	96	13%
Admision for surgery	225	156	31%	175	134	23%
**CRITICAL CARE UNIT (CCU)**						
CCU number	17	36	−112%	15	19	−27%
CCU patients	15	33	−120%	14	17	−21%
(**b**)						
	**CENTER A**			**CENTER B**		
	**Number COVID**	**Number Control**	**%**	**Number COVID**	**Number Control**	**%**
**Number**	3981	3974		3605	3590	
**Primary Care Visits**						
Number of visits	14,801	13,045	12%	14,195	9769	31%
Number of patients	2988	2681	10%	2665	1952	27%
**Specialist Visits**						
Number of visits	2493	2065	17%	2243	1710	24%
Number of patients	1008	885	12%	847	633	25%
**Emergency Room**						
Number of visits	675	486	28%	610	418	31%
Number of patients	468	367	22%	431	315	27%
**Hospital Admissions**						
Number of admissions	62	32	48%	84	57	32%
Number of patients	51	31	39%	72	49	32%
Admision from Emergency	24	7	71%	39	21	46%
Admision scheduled	17	4	76%	26	24	8%
Admision for surgery	21	21	0%	19	12	37%
**CRITICAL CARE UNIT (CCU)**						
CCU number	0	0		1	5	−400%
CCU patients	0	0		1	4	−300%

## Data Availability

The data presented in this study are available in the article and supplementary materials.

## Supplementary Materials

The following supporting information can be downloaded at: https://www.mdpi.com/article/10.3390/jcm12185768/s1, Table S1: (**a**). Number of new diagnostics and patients for each system affected in the study population >17-year-old. in the two Centers. (**b**) Number of new diagnostics and patients for each system affected in the study population <18-year-old in the two Centers.
