# Supplementary material for: Impact of Long-COVID on Health Care Burden: A Case Control Study
Source: J Clin Med. 2023 Sep 5;12(18):5768. doi: 10.3390/jcm12185768 (PMC10531592; doi:10.3390/jcm12185768)
Supplement: Supplementary file 1 [file jcm-12-05768-s001.zip › jcm-2581709-supplementary.docx]

**Supplementary Table S1**

(**a**) Number of new diagnostics and patients for each system affected in the study population >17-year-old in the two Centers. (**b**) Number of new diagnostics and patients for each system affected in the study population <18-year-old in the two Centers

| **(a)** | | | | |
| --- | --- | --- | --- | --- |
|  | **CENTER A** | | **CENTER B** | |
|  | COVID | CONTROL | COVID | CONTROL |
| **DIAGNOSTICS** |  |  |  |  |
| Number | 3843 | 2540 | 4391 | 2404 |
| NEUROPSYCHIATRIC | 928 | 632 | 976 | 526 |
| DIGESTIVE | 667 | 524 | 723 | 441 |
| INFECTIOUS | 437 | 325 | 378 | 229 |
| MUSCULOSKELETIC | 593 | 336 | 616 | 336 |
| DERMATOLOGIC | 449 | 241 | 436 | 225 |
| RESPIRATORY | 410 | 218 | 623 | 224 |
| OTHER | 165 | 122 | 226 | 161 |
| GINECOLOGY | 101 | 84 | 133 | 96 |
| CARDIOVASCULAR | 93 | 58 | 280 | 166 |
| **PATIENTS** |  |  |  |  |
| Number | 3082 | 2104 | 2883 | 1715 |
| NEUROPSYCHIATRIC | 874 | 592 | 779 | 432 |
| DIGESTIVE | 639 | 495 | 582 | 366 |
| INFECTIOUS | 426 | 311 | 315 | 187 |
| MUSCULOSKELETIC | 571 | 330 | 553 | 300 |
| DERMATOLOGIC | 431 | 237 | 382 | 200 |
| RESPIRATORY | 397 | 211 | 451 | 165 |
| OTHER * | 165 | 121 | 196 | 135 |
| GINECOLOGY | 98 | 82 | 118 | 80 |
| CARDIOVASCULAR | 93 | 57 | 200 | 122 |
| **(b)** | | | | |
|  | **CENTER A** | | **CENTER B** | |
|  | COVID | CONTROL | COVID | CONTROL |
| **DIAGNOSTICS** |  |  |  |  |
| Number | 801 | 644 | 839 | 530 |
| NEUROPSYCHIATRIC | 129 | 105 | 165 | 85 |
| DIGESTIVE | 127 | 123 | 174 | 119 |
| INFECTIOUS | 241 | 203 | 167 | 125 |
| MUSCULOSKELETIC | 72 | 41 | 59 | 40 |
| DERMATOLOGIC | 90 | 72 | 73 | 51 |
| RESPIRATORY | 68 | 39 | 90 | 44 |
| OTHER | 33 | 26 | 62 | 39 |
| GINECOLOGY | 34 | 32 | 40 | 20 |
| CARDIOVASCULAR | 7 | 3 | 9 | 7 |
| **PATIENTS** |  |  |  |  |
| Number | 660 | 534 | 584 | 387 |
| NEUROPSYCHIATRIC | 123 | 98 | 127 | 71 |
| DIGESTIVE | 122 | 121 | 134 | 99 |
| INFECTIOUS | 216 | 190 | 143 | 107 |
| MUSCULOSKELETIC | 69 | 40 | 52 | 37 |
| DERMATOLOGIC | 86 | 70 | 68 | 46 |
| RESPIRATORY | 65 | 39 | 73 | 38 |
| OTHER * | 33 | 26 | 53 | 36 |
| GINECOLOGY | 33 | 30 | 36 | 18 |
| CARDIOVASCULAR | 7 | 3 | 7 | 4 |

**Other (' HEMATOLOGIC ',' OFTALMOLOGY ',' ORAL CAVITY ')*
